# Letter to the editor regarding “Trends in lumbar epidural injection selection: A survey of practitioner preferences and practice patterns”

**DOI:** 10.1016/j.inpm.2025.100637

**Published:** 2025-08-27

**Authors:** Parth Aphale, Himanshu Shekhar, Shashank Dokania

**Affiliations:** Dr. D.Y. Patil Vidyapeeth (Deemed to be University), Pimpri, Pune, Maharashtra, India

To the Editor,

We read with interest the article by Triglia et al. titled *“Trends in lumbar epidural injection selection: A survey of practitioner preferences and practice patterns”* [1]. The study provides valuable insights into how provider background and training influence the selection of lumbar epidural steroid injection (ESI) techniques. We commend the authors for conducting a nationwide survey through the International Pain and Spine Intervention Society (IPSIS), which sheds light on prevailing practice patterns. However, we would like to offer several observations that may enhance future studies and improve clinical applicability.

Firstly, while the study recognizes symptomatology—especially radicular patterns—as a dominant driver of approach selection, the scenarios presented were overly simplified and excluded key clinical variables that often influence decision-making. For example, factors such as multilevel disc pathology, bilateral symptoms, prior spine surgery, anticoagulation status, and the presence of spinal instrumentation were not accounted for. These elements frequently dictate the choice between interlaminar and transforaminal approaches, especially in complex cases. The survey design, though pragmatic for response rate, limits extrapolation to real-world practice where procedural nuances are often case-dependent.

Secondly, while the authors highlight the widespread use of dexamethasone in transforaminal ESIs, the continued selection of particulate steroids like triamcinolone—albeit less frequently—is concerning given the associated risks of embolic events and spinal cord infarction. Despite previous FDA advisories and existing guideline recommendations [2]., adherence to best practices remains inconsistent. The finding that even practitioners trained under IPSIS guidelines selected particulate steroids suggests a gap between knowledge dissemination and practical implementation. Incorporating mandatory clinical reasoning responses in future surveys could help elucidate the basis for such decisions and uncover barriers to safer practice patterns.

Additionally, the interpretation of survey results might have benefited from multivariate statistical analysis. While the study reports univariate associations between training type, years in practice, and injection preference, it does not assess the independent predictive value of each variable. Employing regression models could more robustly identify which factors are most strongly associated with guideline-concordant practices. This would inform targeted educational interventions, particularly for clinicians in solo or private practice settings, where deviations from academic norms appear more frequent [3,4].

Moreover, regional variations were not sufficiently explored. Given that practice patterns can differ significantly across healthcare systems and reimbursement models, an analysis stratified by country or state could uncover geographic trends and help tailor regional policy or educational outreach.

Lastly, we advocate for future studies to integrate qualitative methodologies, such as semi-structured interviews or focus groups, to complement quantitative survey data. This mixed-methods approach would provide a richer understanding of how experiential knowledge, institutional culture, and patient preferences influence procedural choices [5].

In conclusion, while the current study provides a foundational understanding of practitioner preferences in lumbar ESI, incorporating more complex clinical contexts, justifications for decision-making, and multivariate analysis could enhance its translational value. We encourage the authors and the broader community to continue refining survey tools to better reflect the intricacies of spine care in contemporary practice.

## CRediT authorship contribution statement

SD- Analysis, Interpretation, Writing the manuscript. PA- Data acquisition, Writing the manuscript. HS- Conception and Design, Final review, Conceptualization.

## Funding

None.

## Declaration of competing interest

The authors declare that they have no known competing financial interests or personal relationships that could have appeared to influence the work reported in this paper.
